# The fluoroquinolone compounds potentiate the antifungal activity of the echinocandins against Aspergillus fumigatus

**DOI:** 10.1042/BSR20250001

**Published:** 2025-02-04

**Authors:** Jin-Ju Choi, Suzie Kang, Yoonseo Lee, Dong-Hyun Lee, Yuju Jang, Ja-Il Goo, Yongseok Choi, Dongho Lee, Cheol-Won Yun

**Affiliations:** 1 School of Life Sciences and Biotechnology, Korea University, Anam-dong, Sungbuk-gu, Seoul, Republic of Korea; 2 EsgelBio Co, Anam-dong, Sungbuk-gu, Seoul, Republic of Korea; 3 Department of Plant Biotechnology, College of Life Sciences and Biotechnology, Korea University, Seoul, Republic of Korea

**Keywords:** *A. fumigatus*, amphotericin B, antifungal drugs, caspofungin, voriconazole

## Abstract

The antifungal drugs of the echinocandin family show high efficacy against *Aspergillus fumigatus*. However, their paradoxical effect, which restores fungal growth at high drug concentrations, and the emergence of resistant strains necessitate improvements. We identified 13 fluoroquinolone compounds from a chemical library containing 10,000 compounds that potentiate the antifungal activity of caspofungin. Among them, NE-E07 significantly enhanced the efficacy of echinocandins against *A. fumigatus*, including resistant strains, without potentiating other antifungal families like voriconazole or amphotericin B. Specifically, NE-E07 demonstrated a unique ability to potentiate caspofungin’s activity against the echinocandin-resistant strain USHM-M0051 isolated from patients. Our experiments revealed that NE-E07, in combination with caspofungin, affected ergosterol biosynthesis in a manner consistent with azole drugs. Docking tests suggest that NE-E07 has a high binding affinity with CYP51, which affects ergosterol biosynthesis similarly to azole drugs. Interestingly, known fluoroquinolones like ciprofloxacin, nalidixic acid, and norfloxacin did not show this potentiating effect, suggesting that NE-E07’s unique structure is critical for its activity. Moreover, NE-E07 did not enhance echinocandin activity against *Candida albicans* or *Cryptococcus neoformans*, highlighting its specific action against *A. fumigatus*. *In vivo* studies demonstrated that co-treatment with NE-E07 and caspofungin increased the survival rate of mice infected with *A. fumigatus*. This significant improvement in survival underscores the potential clinical relevance of NE-E07 as a co-administered drug with echinocandins for treating fungal infections, particularly those resistant to echinocandins.

## Introduction

Fungal infections often not only affect the infection sites such as the skin or fingernails but can also lead to serious and life-threatening conditions such as meningitis or pneumonia [1,2]. The cellular structure of fungi is similar to mammalian cells because fungi are also eukaryotic cells [3], which poses significant challenges in developing fungal-specific drugs. Despite these challenges, several antifungal drugs have been developed and are used to treat and prevent fungal infections. Antifungal drugs are generally classified into three families: azoles, polyenes, and echinocandins, based on their structure or mode of action [4–6]. The azole family works by inhibiting the function of enzymes necessary for synthesizing ergosterol, a critical component of fungal cell membranes, leading to membrane instability and leakage. Common examples include ketoconazole, clotrimazole, fluconazole, and itraconazole. The polyene family binds to ergosterol in the fungal plasma membrane, creating pores that cause the cell to rupture, with amphotericin B and natamycin being notable examples. Echinocandins inhibit the function of Fks1, an enzyme involved in synthesizing β-glucan, an essential component of the fungal cell wall, resulting in cell death. Examples include caspofungin, micafungin, and anidulafungin. Echinocandins are often used for the initial treatment of invasive aspergillosis (IA) due to their relatively low renal and hepatic toxicity and minimal drug interactions.

However, the emergence of antifungal resistance poses a growing threat [7]. Some fungal species, like *Aspergillus fumigatus*, are naturally resistant to specific antifungal drugs such as fluconazole [8]. Resistance is often exacerbated by improper use and prolonged exposure to antifungal drugs, such as reducing dosage, shortening treatment duration, or using subtherapeutic doses [9,10]. The overuse of antibiotics has also led to an overgrowth of *Candida albicans*, a yeast naturally found in the gut [11]. To combat resistant fungal infections, combination therapies have been explored. Co-treatment strategies, such as combining antifungals with antioxidants or other agents, have shown promising results. For instance, combining amphotericin B with methylglyoxal has been reported to significantly increase the survival rate of mice infected with *C. albicans*. Additionally, combining different types of antifungal drugs has been tested to enhance efficacy [12–14].

In this study, we investigated the potential of fluoroquinolone compounds to potentiate the activity of echinocandins. Fluoroquinolones are well-known antibiotics that inhibit bacterial DNA replication but have not been extensively studied for antifungal activity [15–17]. Our findings indicate that fluoroquinolone compounds, when combined with echinocandins, significantly enhance antifungal activity and improve survival rates in mouse models of fungal infection. Echinocandins are lipopeptides consisting of cyclic hexapeptides N-linked to a fatty acyl side chain and inhibit (1,3)-β-D-glucan synthesis, resulting in fungal cell wall instability [18]. Because there are no mammalian homologs of the fungal cell wall, the side effects of echinocandins are usually mild. Also, echinocandins are neither inducers nor inhibitors of cytochrome P450 isoenzymes [19,20], and few drug interactions are reported [21]. However, the recent emergence of fungi resistant to echinocandins has become a problem, and the resistance to echinocandins is associated with mutations in the gene that encodes a sequence of unique amino acids of Fks protein, which is the putative binding domain for echinocandins [22,23].

These results suggest that fluoroquinolones could be valuable as co-administered drugs in treating invasive fungal infections, especially those caused by resistant strains.

## Results

### Fluoroquinolone derivatives potentiate antifungal effect with caspofungin against *A. fumigatus*

Most antifungal drugs have various side effects and some of which are fatal in host. Caspofungin is an antifungal drug of the echinocandin family and inhibits fungal cell wall synthesis by interfering with β-glucan biosynthesis. Although the echinocandin family is a moderate antifungal drug, it has a paradoxical effect at high concentrations and shows fungistatic function [24]. To discover the drugs that potentiate the activity and remove their paradoxical effect of caspofungin against *A. fumigatus*, we performed a new drug screening using a chemical library provided by the Korea Chemical Bank (https://chembank.org). The drug screening was performed as follows: we first inoculated 1 × 10^4^ conidia into 200 μl of CM broth medium and then treated the culture with approximately 8000 compounds at a concentration of 6.25 μM each, along with caspofungin at 0.5 mg/ml. The cultures were incubated at 37°C for three days, after which we observed the growth of *A. fumigatus*. We identified wells where no growth was observed and selected the corresponding compounds. Next, we conducted two simultaneous experiments. In the first experiment, we treated the plate with the compound alone at 6.25 μM, and in the second, we treated the plate with the compound at 6.25 μM along with caspofungin at 0.5 μg/ml. From this comparison, we identified 13 compounds where growth was observed with the compound alone but no growth occurred when the compound was combined with caspofungin, and we proceeded with these compounds for further experiments. They were fluoroquinolone compounds (Table 1), and we selected one compound (NE-E07) with the best antifungal activity among them for further research.

**Table 1:** NE-E07 has a synergic effect with echinocandins against *A. fumigatus*

	Structure		Structure
Caspofungin	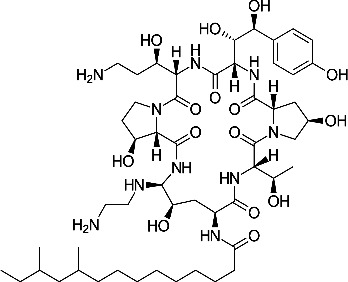	NE-B10	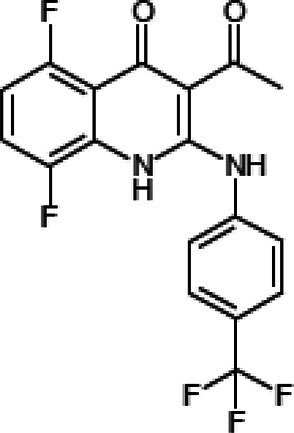
NE-H08	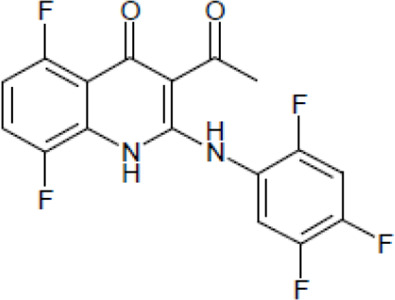	NE-C04	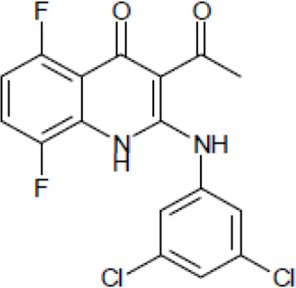
NE-C11	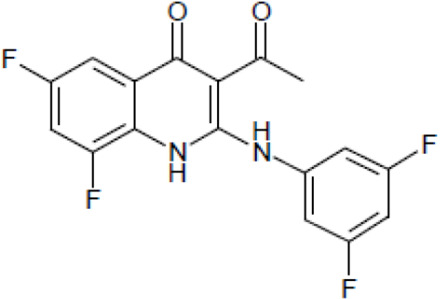	NE-H07	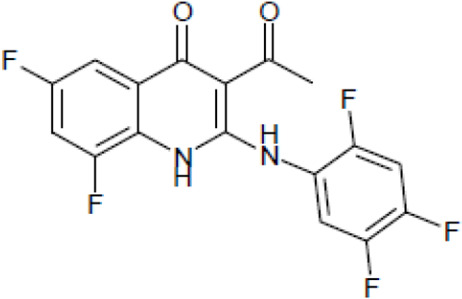
NE-H04	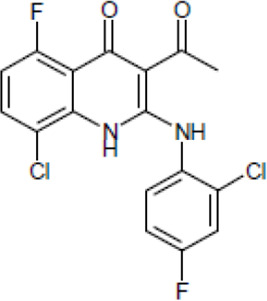	NE-H04	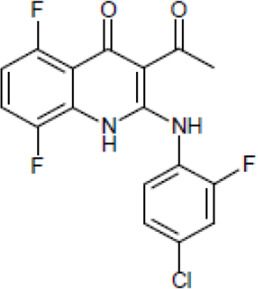
NE-D10	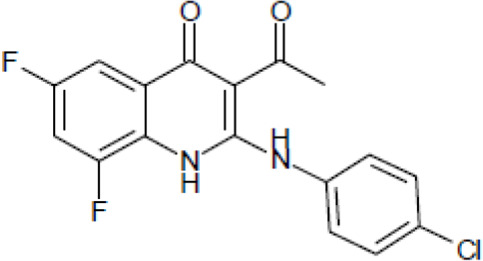	NE-F07	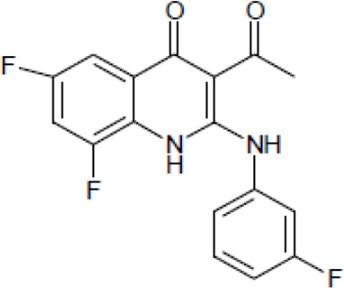
NE-H12	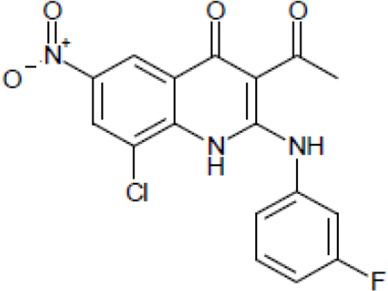	NE-B04	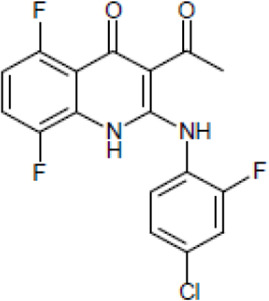
NE-A06	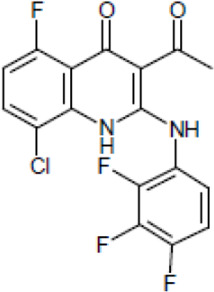	NE-E07	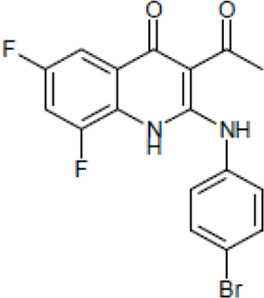

**Figure 1:**
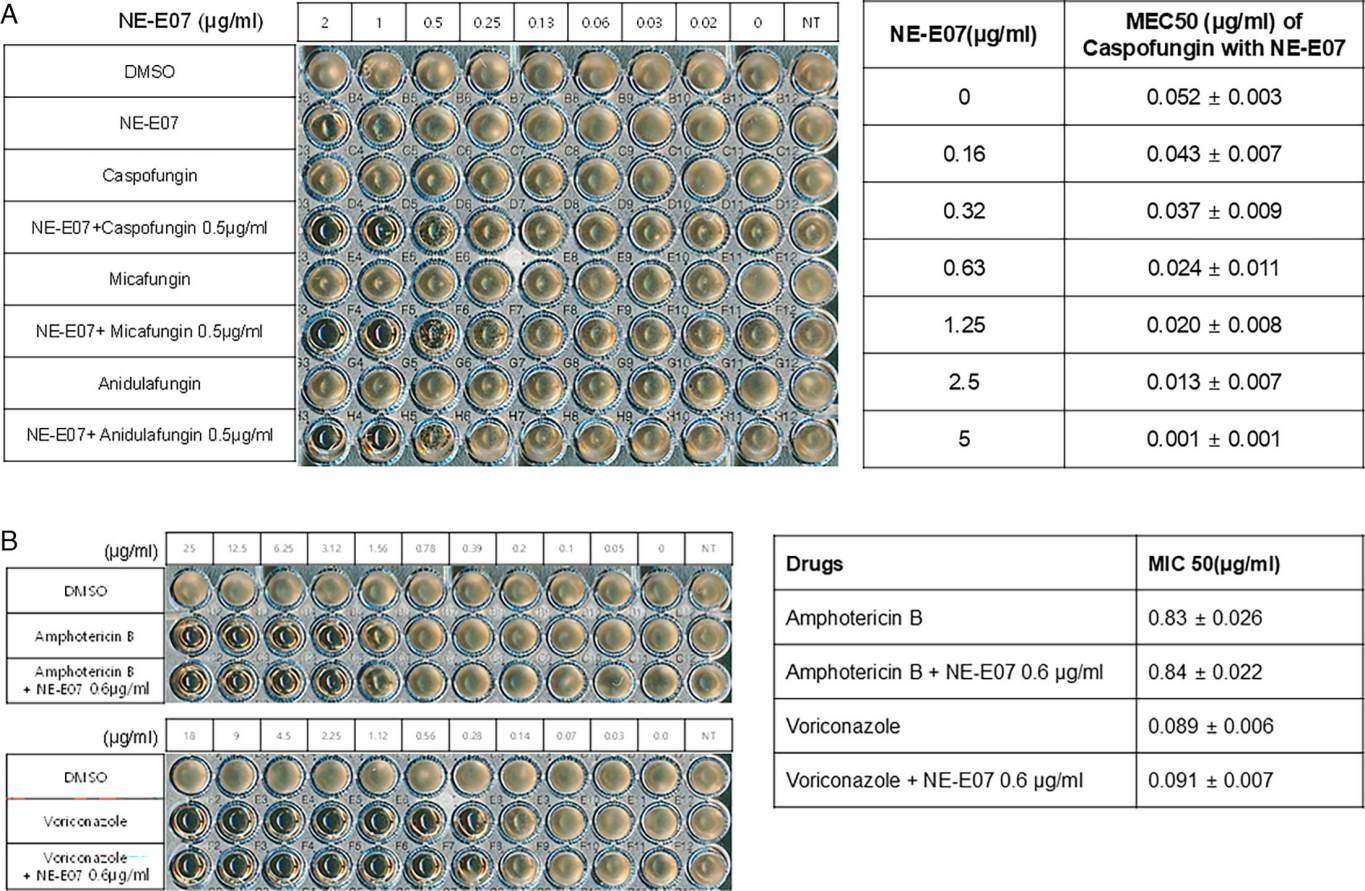
NE-E07 potentiates the activity of echinocandins against *Aspergillus fumigatus*. **(**A**)** 1 × 10^4^/ml of *A. fumigatus* conidia was inoculated to each well containing RPMI broth media in 96-well plates. DMSO is negative control. Caspofungin, micafungin, and anidulafungin were added to the wells with the indicated concentration. NE-E07 was added to the wells from 2 μg/ml to 0 by serial dilution Tto investigate whether NE-E07 potentiates the activity of echinocandins. To measure the MEC50 of the indicated drugs, the resazurin assay was performed as described in Materials and Methods, and each MEC50 is shown in the right panel. **(**B**)** NE-E07 did not potentiate the activity of amphotericin B or voriconazole. The right panel shows the MIC50 when the cells were treated with amphotericin B or voriconazole alone and co-treated with NE-E07 at 0.6 μg/ml.

NE-E07 has a typical fluoroquinolone compound, and to compare the antifungal activity of NE-E07 with other known antifungal drugs, we tested the antifungal activity in the presence or absence of caspofungin. As shown in Table 2, we tested representative antifungal drugs from three families: amphotericin B, voriconazole, and caspofungin. They belong to the polyene, azole, and echinocandin classes, respectively. The MIC50 values of amphotericin B, voriconazole, and MEC50 of caspofungin were 0.83 μg/ml, 0.089 μg/ml, and 0.052 μg/ml, respectively. Interestingly, the MEC50 value of caspofungin was decreased to 0.024 μg/ml when treated with NE-E07 of 0.63 μg/ml, even though the MIC50 value of NE-E07 alone was 2.78 μg/ml as shown in Table 2. The MEC50 value of caspofungin was decreased in a concentration-dependent manner of NE-E07. To determine whether this activity of NE-E07 also works against other echinocandin antifungal drugs besides caspofungin, we examined its effect on the activity of the other echinocandin antifungal drugs micafungin and anidulafungin, as shown in Figure 1A. The results showed that micafungin and anidulafungin showed the same antifungal activity against *A. fumigatus* when treated alone, as does caspofungin, but this paradoxical effect was abolished when treated with NE-E07. NE-E07 potentiated the activity of other antifungal drugs of echinocandins such as that of caspofungin. These results suggest that NE-E07 potentiates the activity of echinocandin-based antifungal drugs.

**Table 2:** NE-E07 has a synergic effect with echinocandins against *A. fumigatus*

Antifungal drugs	Molecular weight	Structure	MIC50 (mg/ml)	MEC50 (mg/ml)
Amphotricin B	924	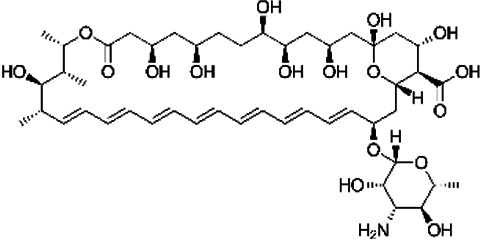	0.83 ± 0.026	−
Voriconazole	349	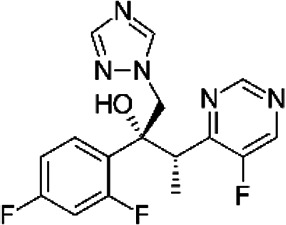	0.089 ± 0.006	−
Caspofungin	1093	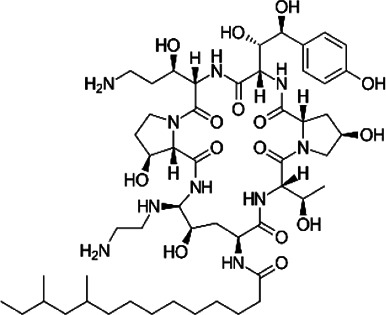	−	0.052 ± 0.003
NE-E07	393.2	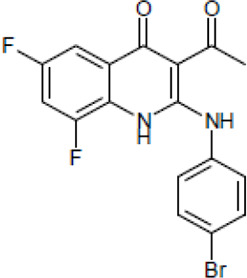	2.78 ± 0.51	−

To determine whether NE-E07 potentiates the activity of echinocandins and other antifungal drug families, we measured MIC50 value of amphotericin B and voriconazole with or without NE-E07. As shown in Figure 1B, cells were treated with the indicated concentrations of amphotericin B and voriconazole, and the MIC50 values of amphotericin B and voriconazole were 0.83 μg/ml and 0.089 μg/ml, respectively, when treated alone. The MIC50 values of amphotericin B and voriconazole were not changed, even though 0.6 μg/ml NE-E07 was treated together. These results suggest that NE-E07 does not potentiate the activity of amphotericin B and voriconazole and that NE-E07 works on the activity of the echinocandins.

### NE-E07 potentiates the antifungal activity of caspofungin against the echinocandin-resistant strain of *A. fumigatus* USHM-M0051.

**Figure 2:**
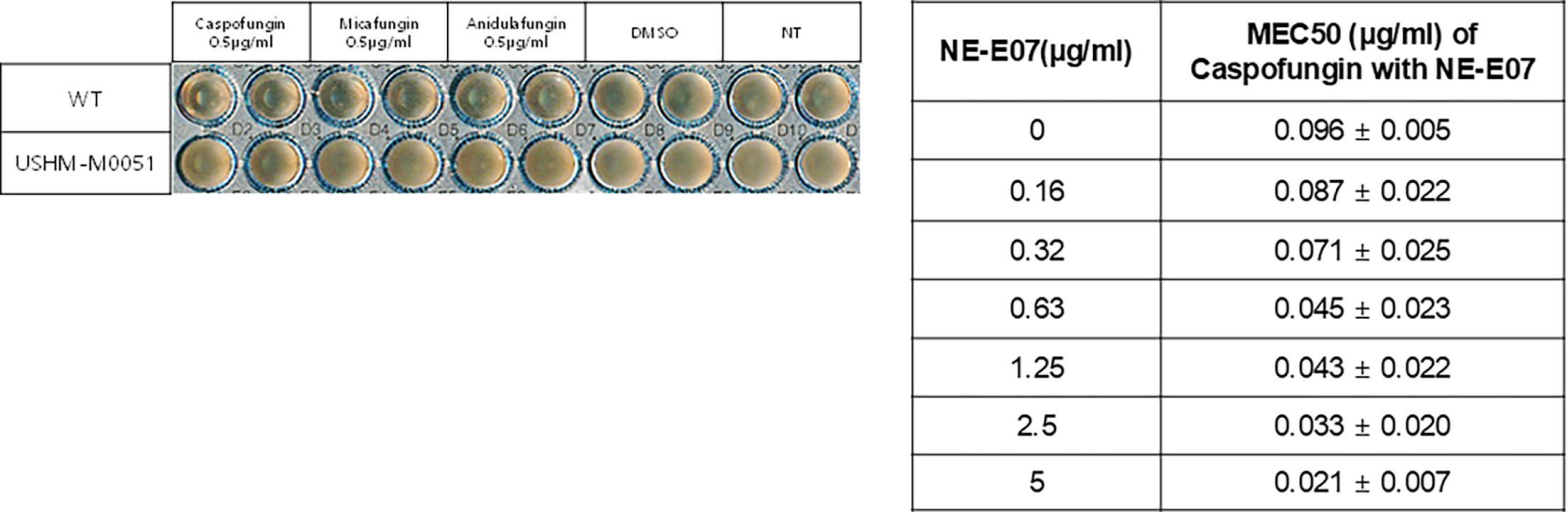
NE-E07 potentiates the activity of echinocandins against echinocandin-resistant strain USHM-0051. NE-E07 potentiates the activity of caspofungin and is active against the echinocandin-resistant strain USHM-0051. 1 × 10^4^/ml of echinocandins -resistant strain USHM-0051 conidia was inoculated in RPMI broth media containing the indicated concentration of caspofungin, micafungin, and anidulafungin in 96-well plates. DMSO is negative control. The MEC50 of the co-treatment of NE-E07 and caspofungin was measured by the resazurin assay. The MEC50 was reduced when NE-E07 was co-treated with caspofungin.

The echinocandin-resistant strain of *A. fumigatus* USHM-M0051 was isolated from the patient’s sputum and kindly provided by the National Culture Collection for Pathogens, Korea (https://nccp.kdca.go.kr). To confirm the resistance of USHM-M0051 to caspofungin, we cultured wild-type and USHM-M0051 cells in 96-well plates with or without 0.5 μg/ml of caspofungin. As shown in Figure 2, USHM-M0051 showed no growth defects with 0.5 μg/ml of caspofungin, although wild-type *A. fumigatus* showed slightly impaired growth with 0.5 μg/ml of all echinocandin antifungal drugs. We then examined the effects of NE-E07 on USHM-M0051 when treated with caspofungin by measuring MEC50. NE-E07 was treated to the wild-type and USHM-M0051 cells with 0.5 μg/ml of caspofungin or alone. The MEC50 value of USHM-M0051 was decreased from 0.096μg/ml to 0.021μg/ml of caspofungin as shown in Figure 2B. These results indicate that the co-treatment of NE-E07 and caspofungin strongly potentiates the antifungal activity of caspofungin, and it gives a better way to control echinocandin-resistant strains by co-treatment.

**Figure 3:**
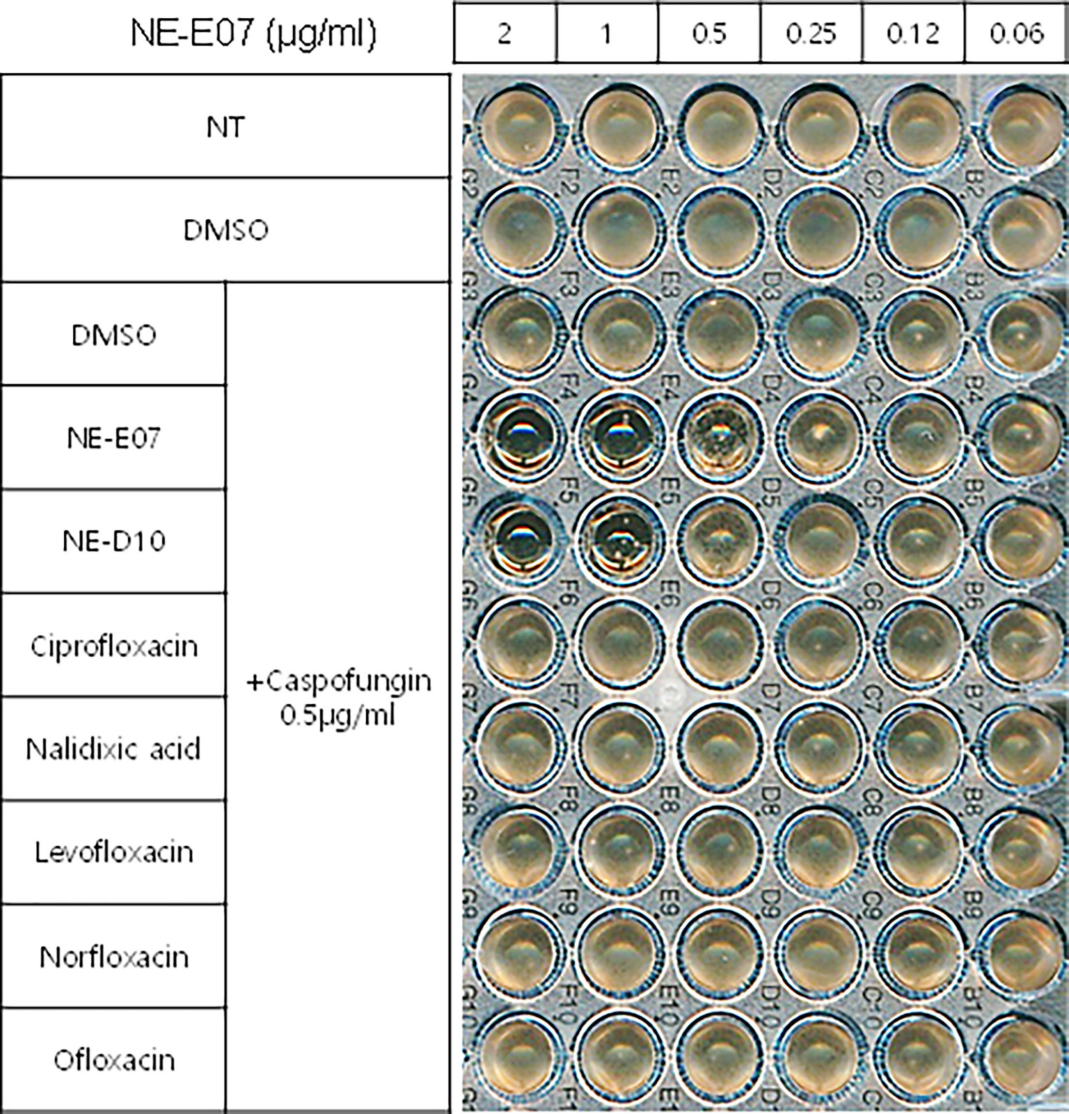
Fluoroquinolone antibiotics does not potentiate the activity of the caspofungin. NE-E07, NE-D10, and five commercial fluoroquinolone antibiotics (ciprofloxacin, nalidixic acid, levofloxacin, norfloxacin, and ofloxacin) were diluted to the indicated concentrations. Each dilution was then treated with 0.5 µg/ml of caspofungin in Thethe wells. The samples were incubated for three days to observe the effects on fungal growth.

We also investigated the antifungal effect of NE-E07 against *C. albicans* and *C. neoformans. C. albicans* and *C. neoformans* were spotted on the YPD plate containing NE-E07 and caspofungin at the indicated concentrations. *C. albicans* was quite sensitive to caspofungin, and no growth was observed at 0.04 μg/ml caspofungin as shown in Supplementary Figure S1. However, there was no enhanced growth defect by NE-E07 against *C. albicans* co-treatment with caspofungin. On the other hand, NE-E07 itself has an antifungal activity against *C. neoformans* as shown in Figure 3B. The addition of 2.5 μg/ml NE-E07 resulted in growth defects of *C. neoformans*. Interestingly, *C. neoformans* is quite resistant to caspofungin and showed the same antifungal activity of NE-E07 alone without enhanced activity even when co-treated with caspofungin. These results suggest that NE-E07 does not potentiate the activity of caspofungin against *C. albicans* and *C. neoformans*.

**Figure 4:**
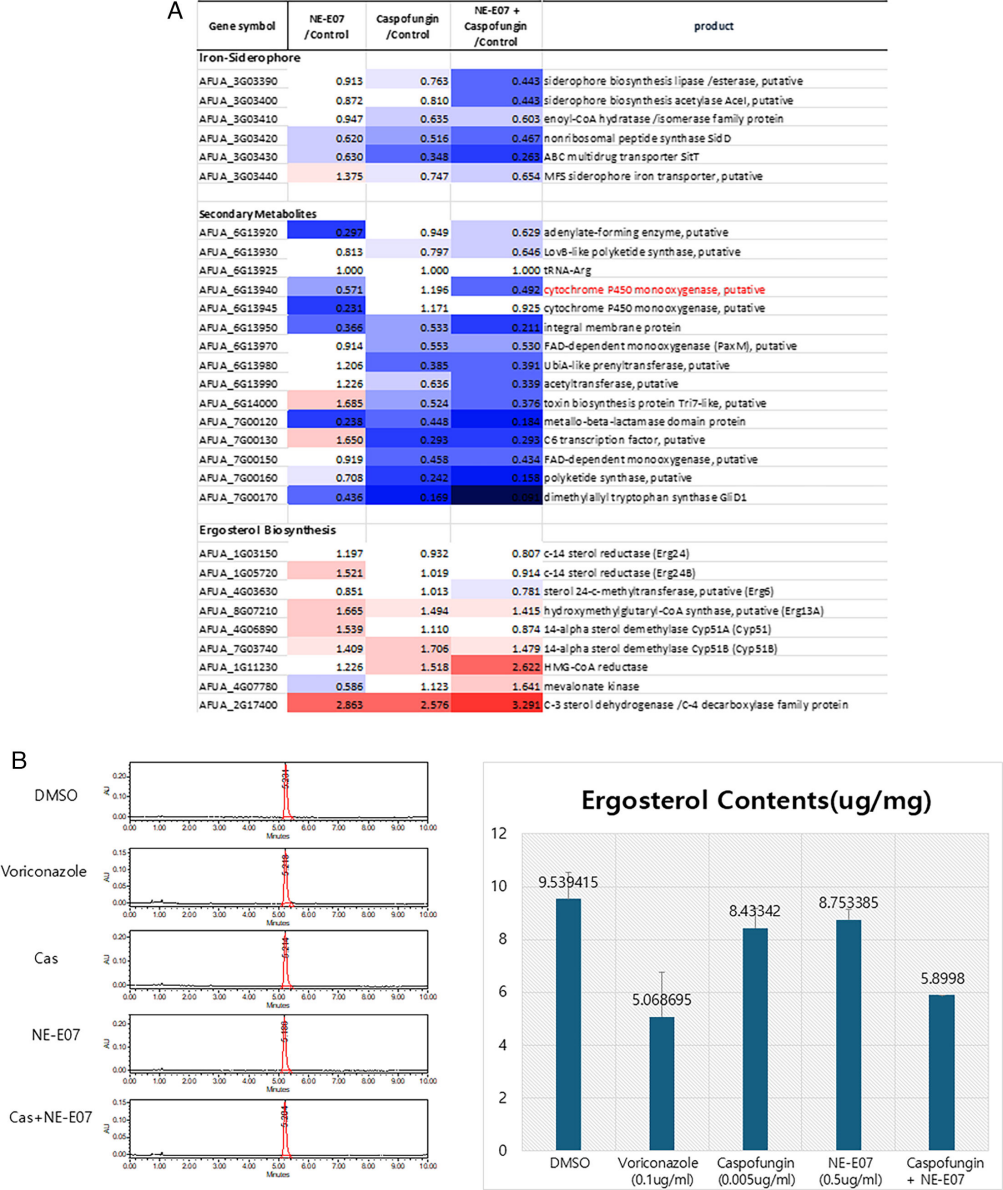
NE-E07 may have a similar function to azole antifungal drug. **(**A**)** RNA-seq analysis on *A. fumigatus* treated with 1 μg/ml NE-E07 and 0.5 μg/ml caspofungin was performed. Total RNA was extracted from the drug-treated cells and RNA-seq analysis was performed. (**B**) Cellular ergosterol was extracted as described in Materials and Methods and analyzed and quantified by HPLC. DMSO was used as a negative control and voriconazole as a positive control.

We then investigated whether quinolone antibiotics potentiate the activity of caspofungin since quinolone antibiotics have a similar structure to NE-E07, which we studied. As shown in Figure 4, NE-E07, NE-D10, and five fluoroquinolone antibiotics were treated in the cells at the indicated concentrations and incubated for three days. NE-D10 (Table 1) is another fluoroquinolone derivative that we screened, and it potentiated the activity of caspofungin against *A. fumigatus*. NE-E07 and NE-D10 potentiated the antifungal activity of caspofungin as shown above. However, all quinolone antibiotics we tested, such as ciprofloxacin, nalidixic acid, levofloxacin, norfloxacin, and ofloxacin, did not appear to potentiate the activity of caspofungin, as shown in Figure 3. These results suggest that all fluoroquinolones do not potentiate the activity of caspofungin and that, specifically, the co-administration of NE-E07 and its derivatives may improve the treatment of patients with fungal infections.

### NE-E07 may have a similar mode of action to azole antifungal drug

We then sought to identify the mode of action of NE-E07 on *A. fumigatus*. We conducted mRNA-seq analysis to identify the mode of action of NE-E07. We understand that analyzing changes in gene expression after antifungal treatment may not be ideal for directly determining the drug’s mode of action. However, examining similarities between the gene expression changes induced by NE-E07 and those caused by other known antifungal drugs could provide valuable clues regarding NE-E07’s mode of action. Initially, we cultured *A. fumigatus* and treated the cultures with either 1 mg/ml NE-E07 or 0.5 μg/ml caspofungin individually. Additionally, we prepared another sample with a combination of 1 μg/ml NE-E07 and 0.5 μg/ml caspofungin. Total RNA was extracted from all samples, and gene expression changes were subsequently analyzed to investigate the effects of these treatments (Figure 4A). From the RNA-seq analysis, we observed that when NE-E07 and caspofungin were co-administered, the expression of genes involved in secondary metabolite biosynthesis, iron metabolism, and ergosterol biosynthesis showed patterns highly similar to those observed when cells were treated with itraconazole and isaconazole [25]. This gene expression profile suggests that the combination of NE-E07 and caspofungin might share a similar mechanism of action (MOA) with azole-type antifungal agents.

To further confirm the mode of action of NE-E07, we measured the ergosterol content of NE-E07-treated cells. As shown in(Figure 4B) *A. fumigatus* was treated with voriconazole, caspofungin, and NE-E07 at the indicated concentrations, and ergosterol was extracted as described in the Materials and Methods. We found that a single peak showing ergosterol was obtained from the DMSO-treated cells as a control, and ergosterol was found and quantified in all samples. Caspofungin- and NE-E07-treated cells showed the same amount of ergosterol as DMSO-treated cells. However, approximately half the amount of ergosterol was found in voriconazole-treated cells. Interestingly, we found that the same level of ergosterol was identified from the caspofungin and NE-E07 co-treated cells as from the voriconazole-treated cells. These results suggest that NE-E07 acts as an azole antifungal drug when co-treated with caspofungin, and we should further find the reason why co-treatment of caspofungin and NE-E07 affects ergosterol biosynthesis.

### NE-E07 is a potential inhibitor of CYP51 from docking study

**Figure 5:**
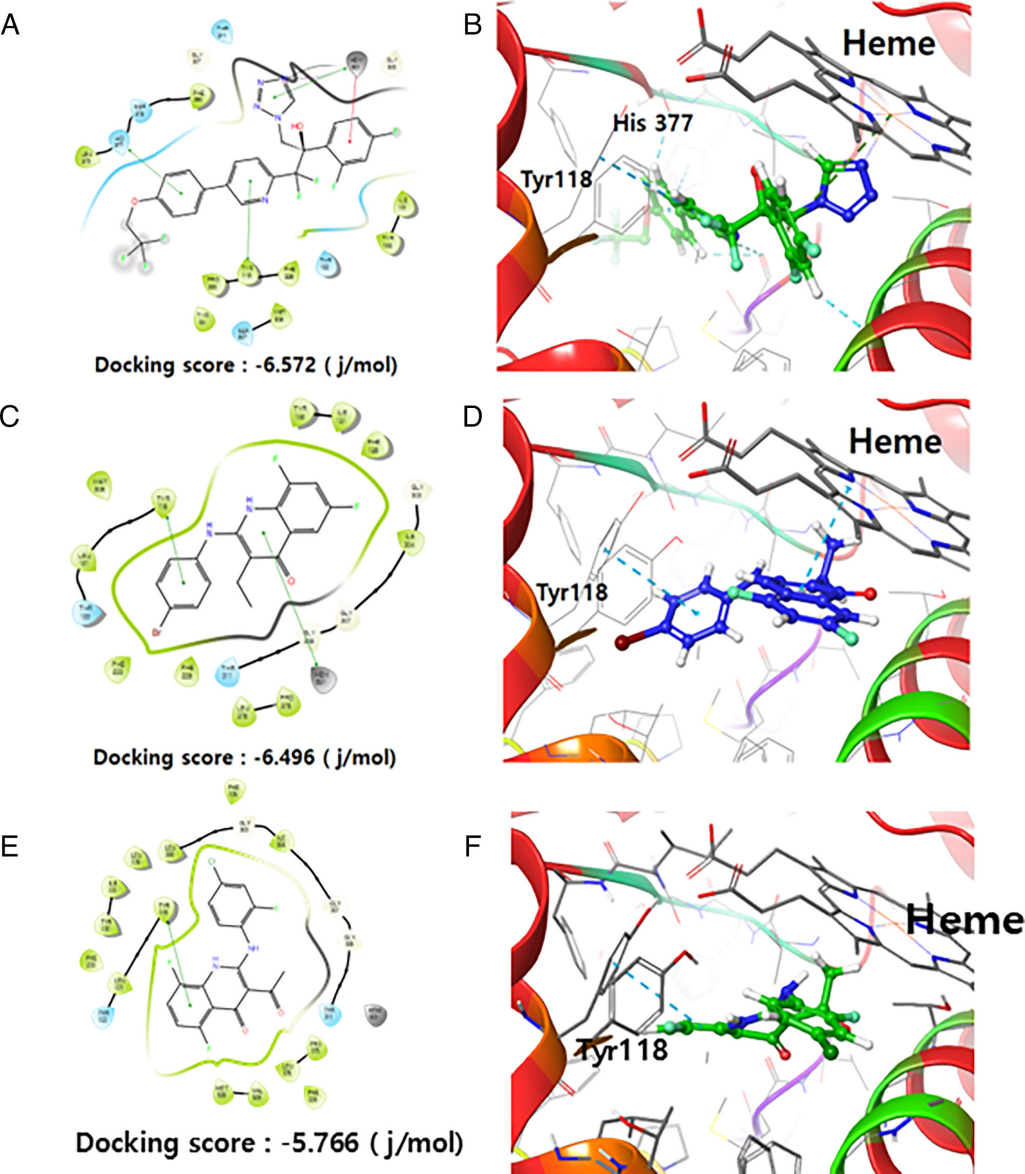
The binding of VT-1161 and NE-E07 involves multiple structural interactions within the active site of CYP51. **(**A**)** VT1161 ligand interaction 2D diagram and docking score. **(**B**)** dDocking study results of VT1161 binding to the active site of CPY51 (Ggreen). **(**C**)** NE-E07 ligand interaction 2D diagram and docking score. **(**D**)** dDocking study results of E07 binding to the active site of CPY51 (Bblue). **(**E**)** NE-B04 ligand interaction 2D diagram and docking score. **(**F**)** dDocking study results of NE-B04 binding to the active site of CPY51 (Bblue).

Analysis of the binding modes of VT1161 and E07 to CYP51 indicated that they bind to the active site and interfere with the catalytic mechanisms of the enzyme. As shown in Figure 5a and b, VT1161 is the known specific inhibitor of CYP51 and it has three π-π stacking interactions identified at the heme active site of CYP51. In particular, π-π staking interactions significantly enhance the binding affinity and stability of heme-ligand complexes by providing additional stabilization energy and specificity through aromatic ring interactions. Several key structural interactions characterize the binding of VT-1161 to the active site of CYP51. VT-1161 forms critical interactions with histidine residues (His377), which play an important role in stabilizing the heme group and ensuring proper alignment with the heme iron, thereby inhibiting enzyme activity. The inhibitor is further stabilized by hydrophobic interactions with amino acid residues surrounding the heme pocket, increasing its binding affinity and preventing displacement. In addition, hydrogen bonds between VT-1161 and specific residues within the active site provide additional stability to the inhibitor-enzyme complex, ensuring a strong and precise binding conformation that effectively inhibits CYP51. Based on the docking studies, NE-E07 showed comparable patterns to VT-1161. NE-E07 exhibited two π-π stacking interactions and confirmed binding to the heme structure (Figure 5c and d). Both ligands shared common π-π staking interactions with Heme and Tyr118, with docking scores similar to VT1161. These results suggest that E07 matches the docking behavior of VT1161, indicating that E07 also possesses substantial inhibitory activity. Additionally, we conducted a docking study of CYP51 with NE-B04, which is structurally similar to NE-E07 but exhibits relatively lower activity (Figure 5e and f). NE-E07 exhibited π-π stacking interactions with Heme, whereas compound NE-B04 showed no binding with Heme. Despite the structural similarities between NE-E07 and NE-B04, the docking results indicate different binding orientations.

**Figure 6:**
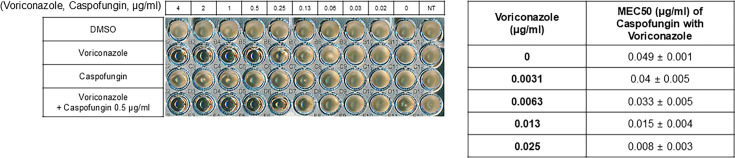
Voriconazole potentiates the activity of echinocandins against *Aspergillus fumigatus*. 1 × 10^4^/ml of *A. fumigatus* conidia was inoculated to each well containing RPMI broth media in 96-well plates. DMSO is negative control. 0.5 μg/ml of caspofungin was added to the wells and voriconazole was added to the wells with the indicated concentration and from 4 μg/ml to 0 by serial dilution Toto investigate whether voriconazole potentiates the activity of echinocandins. To measure the MEC50 of the indicated drugs, the resazurin assay was performed as described in Materials and Methods and each MEC50 is shown in the right panel.

**Figure 7:**
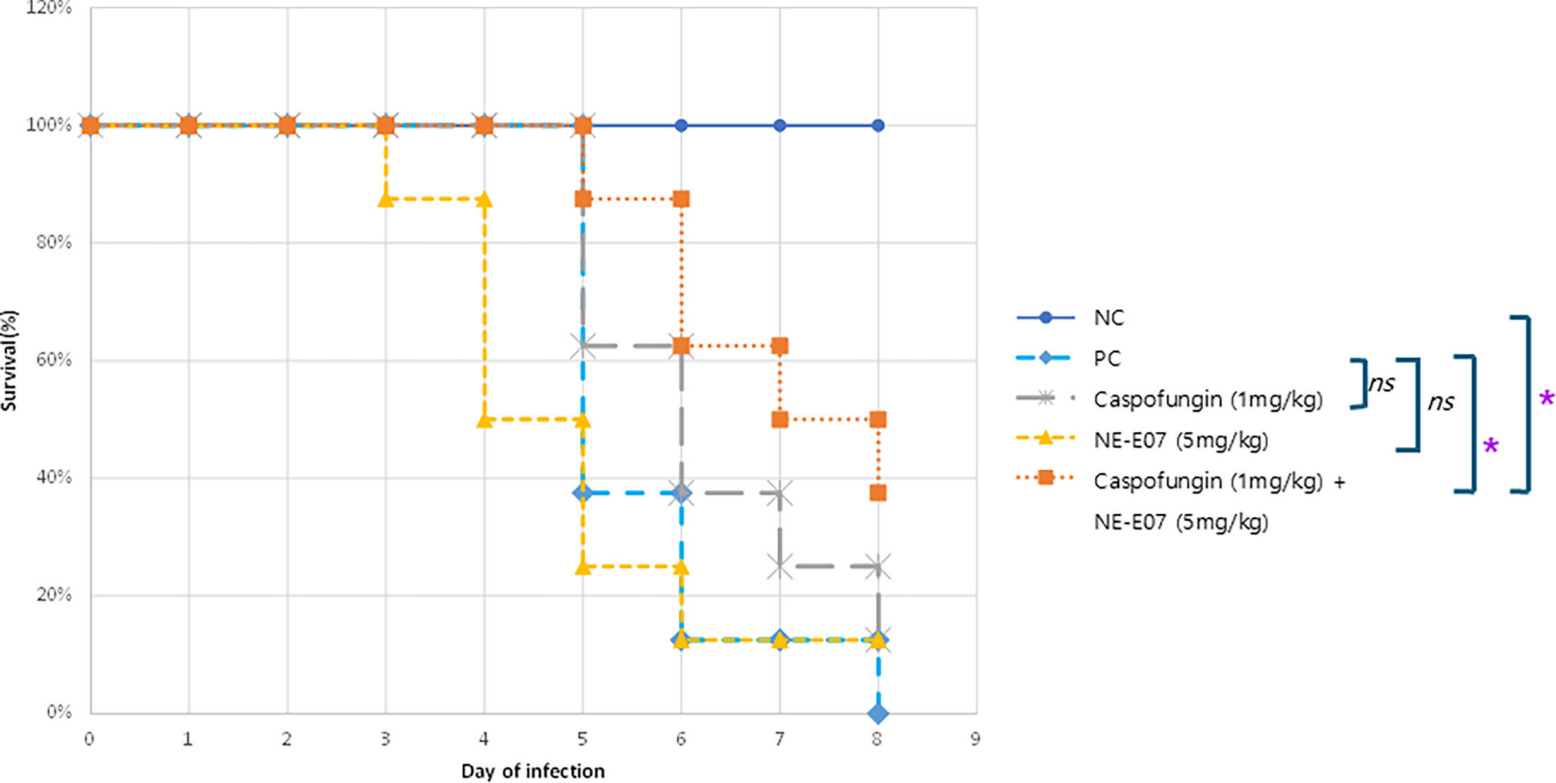
Co-administration of caspofungin and NE-E07 increases the survival rate of mice infected with *Aspergillus fumigatus*. A murine infection assay was performed to investigate the effect of co-administration of caspofungin and NE-E07 in Micemice. Mice were anaesthetized by isoflurane inhalation and treated intranasally with a 20-μl drop of a solution containing 5 × 10^5^ conidia. A drop of the spore solution was placed on the nose of the anaesthetized mice. There were eight mice in each experimental group, and two independent experiments were performed. After 24 hours of infection, 1 mg/kg of caspofungin and 5 mg/kg of NE-E07 were administered orally daily, alone, or both. NC is negative control without infection of *A. fumigatus* and drugs. PC indicates infection of *A. fumigatus* without drug treatment. *: *P*<0.05 indicates a significant difference compared towith the control group. *ns* indicates no significant differences compared towith the control group.

Our results indicate that NE-E07 works as an azole antifungal drug, and we checked whether other azole antifungal drugs also potentiate the activity of caspofungin. It has been reported that voriconazole treatment enhances the antifungal activity of caspofungin in mouse infection experiments [26]. As shown in Figure 6, the effect of voriconazole on caspofungin activity was tested and resazurin assay was also performed to obtain the MEC50. The cells were incubated in the RPMI media, and the indicated drugs were added to the cells. As shown in Figure 7, the co-treatment of caspofungin and voriconazole showed higher antifungal activity than either treatment alone or the MEC50 of caspofungin was 0.049 μg/ml when treated alone. However, the MEC50 of caspofungin when treated with voriconazole was 0.033 μg/ml, and this result indicates that the azole family may potentiate the activity of echinocandins.

### Co-administration of caspofungin and NE-E07 increases the survival rate of mice infected with *A. fumigatus*

**Figure 8:**
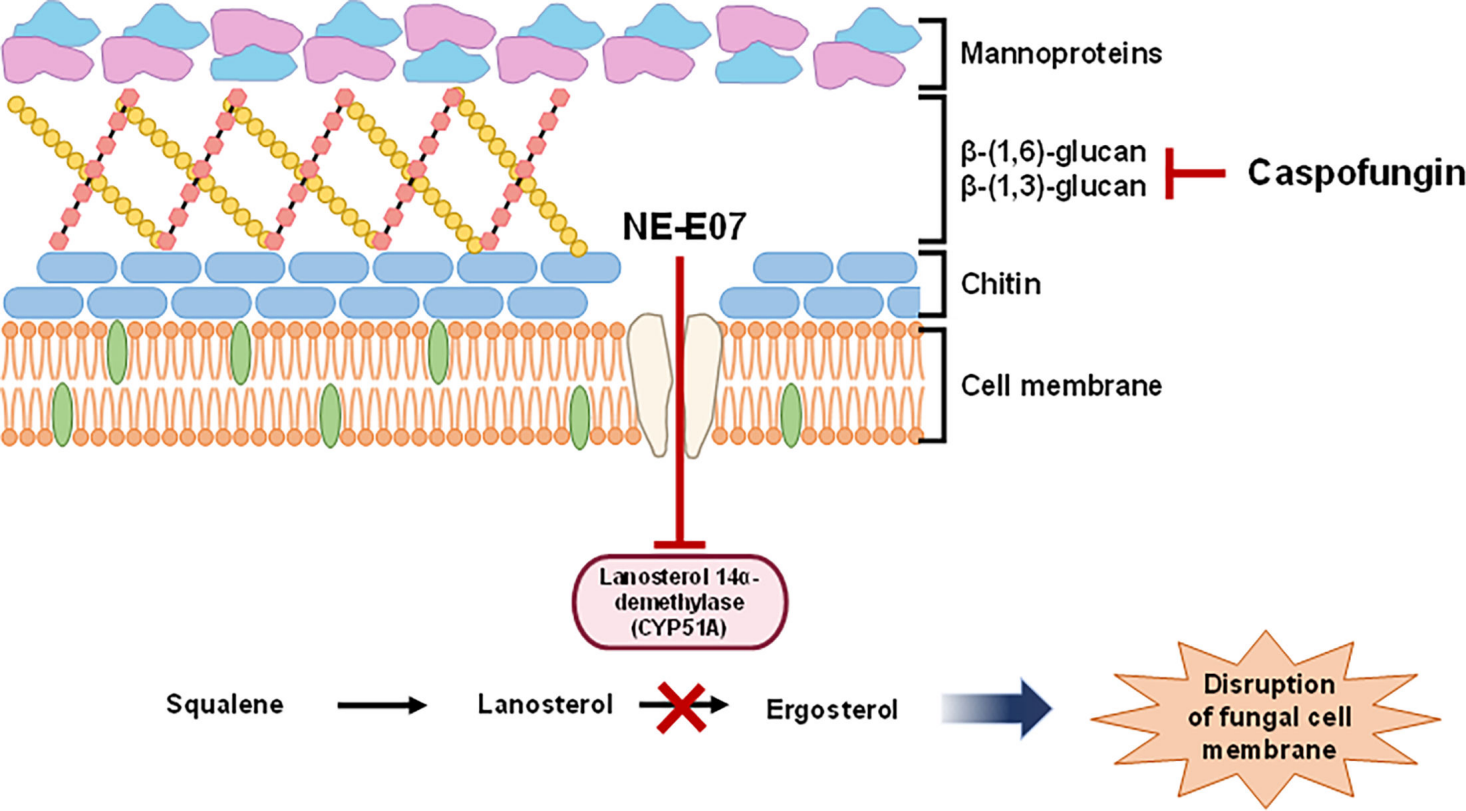
The hypothetical mode of action of NE-E07 with caspofungin.

A murine infection assay was performed to investigate the effect of co-administration of caspofungin and NE-E07 in mice. Six-week-old male BALB/c mice were obtained from Doo-Yeol Biotech Co. (Seoul, Korea). Starting on day 4, mice were injected with cyclophosphamide (150 mg/kg body weight) once every three days to suppress their immune system, and hydrocortisone acetate (112.5 mg/kg body weight) was injected on day 1. Mice were anesthetized by isoflurane inhalation and treated intranasally with a 20-µl drop of a solution containing 5 × 105 conidia. A drop of the spore solution was placed on the nose of the anesthetized mice, allowing the mice to inhale the solution without pain. There were eight mice in each experimental group, and two independent experiments were performed. After 24 hours of infection, 1 mg/kg caspofungin and 5 mg/kg NE-E07 were administered orally daily, alone, or both, as shown in Figure 7. When only one drug was administered, 20% hydroxypropyl-β-cyclodextrin (HP-β-CD), the solvent used to dissolve drugs, was administered instead of the drug. As shown in Figure 8, the mice infected with *A. fumigatus* started to die four days after infection; although NE-E07 was administered, seven mice died after six days of infection, and 12.5% of the mice survived eight days after infection. Mice that administered no drug after infection started to die after six days, and 0% of the mice survived after eight days. Caspofungin-treated mice began to die after six days of infection, and 12.5% of the mice survived eight days after infection. Finally, both caspofungin- and NE-E07-treated mice started to die after six days, and 37.5% of the mice survived eight days after infection. This result confirmed that NE-E07 potentiates the antifungal activity of caspofungin.

## Discussion

IA is a severe fungal infection caused by *A. fumigatus*, which is particularly lethal to immunocompromised patients [27]. Current antifungal drug treatments for IA include azoles, polyenes, and echinocandins [28–30]. Among these, echinocandins are preferred for initial therapy due to their relatively low renal and hepatic toxicity and minimal drug interactions [31,32]. Echinocandins work by inhibiting cell wall synthesis, specifically targeting β-glucan synthesis, making them effective as both salvage treatment and prophylactic therapy. However, echinocandins are classified as fungistatic, and resistance is increasing, primarily due to mutations in the *Fks1* gene, which lead to structural changes in the Fks1 protein [33].

In this study, we screened a new fluoroquinolone compound, NE-E07, that potentiates the activity of echinocandins. NE-E07 demonstrated enhanced efficacy in inhibiting the growth of *A. fumigatus* when used in combination with echinocandins, as confirmed by our mouse infection model experiments (Figure 8). Co-treatment with NE-E07 and caspofungin significantly increased the survival rate of infected mice compared with caspofungin alone, although NE-E07 by itself was ineffective. These findings suggest that NE-E07 could serve as a valuable co-administered drug with caspofungin.

The mode of action of NE-E07 as an antifungal drug is an important issue that needs to be addressed. To explain the MOA of NE-E07, we performed RNA sequencing analysis (RNA-seq) and RNA-seq revealed that NE-E07-treated cells exhibited a gene expression profile similar to that of itraconazole or isaconazole [25]. This suggests that NE-E07 may function similarly to azole antifungal drugs. Further supporting this hypothesis, high-performance liquid chromatography (HPLC) analysis showed that NE-E07, in combination with caspofungin, affected ergosterol biosynthesis in a manner consistent with azole drugs, but this effect was not observed in cells treated with NE-E07 alone. NE-E07 is hypothesized to interact with CYP51 (lanosterol 14-α demethylase), an enzyme involved in the biosynthesis of ergosterol, a critical component of the fungal cell membrane. Docking tests also suggest that NE-E07 has a high binding affinity for CYP51, similar to azole antifungal drugs, which supports this proposed mode of action (Figure 8). This interaction disrupts the production of ergosterol, leading to cell membrane instability and cell death.

Given NE-E07’s potential similarity to azole antifungal drugs, we investigated whether other azole drugs could also potentiate the activity of echinocandins. Several reports indicate that co-administration of the azole antifungal voriconazole and caspofungin increases the survival rate in mice from the murine infection experiments [34,35]. Additionally, it is reported that co-administration of voriconazole and caspofungin might be considered preferable therapy for organ transplant recipients with IA [36]. These reports support our findings and suggest that such combination therapies could be beneficial in improving treatment outcomes for fungal infections. Our data also confirmed that the antifungal effect is enhanced when voriconazole and caspofungin are administered simultaneously (Figure 6).

However, in previously reported studies on the combined treatment of voriconazole and caspofungin, each antifungal drug exhibited high antifungal activity even when used alone [34]. However, in our study, NE-E07 alone did not show significant antifungal activity. Despite NE-E07 being structurally different from traditional antifungal drugs as a fluoroquinolone, it appears to interact with CYP51, as evidenced by its inhibition of ergosterol synthesis. This supports its potential MOA similar to that of azole antifungal drugs. We propose a hypothetical mode of action for NE-E07 in combination with caspofungin (Figure 8). Our findings indicate that caspofungin may compromise the integrity of the cell wall and enhance NE-E07’s proximity to CYP51, the target of NE-E07, thereby boosting its antifungal activity. Due to the rigid cell wall structure of fungi, it is difficult for substances to pass through. To overcome this structural barrier, damaging the cell wall can be an effective method to enhance the passage of compounds through the cell wall. These mechanisms are observed with echinocandins and have also been reported in the case of amphotericin B [5]. Amphotericin B interacts with ergosterol and creates pores or channels within the membrane, causing leakage of cellular contents and ultimately leading to cell death. This pore-forming action enhances the permeability of the cell membrane, making it easier for other drugs to enter the cell.

In conclusion, NE-E07, a fluoroquinolone derivative, shows promise as a co-administered drug with caspofungin for treating IA. Fluoroquinolone antibiotics, such as ciprofloxacin, delafloxacin, levofloxacin, and norfloxacin, are already widely used and effective against bacterial infections [37–40]. Our findings suggest that NE-E07 and specific fluoroquinolone antibiotics could be beneficial in future clinical trials targeting fungal infections. Further studies are necessary to validate these results and fully understand the clinical implications of NE-E07.

## Materials and methods

### Aspergillus strain, media, and conidia spore preparation

The fungal strain used in this study was *A. fumigatus* A1163. The cells were cultured at 37°C in *Aspergillus* minimal medium (AMM; 1% glucose, 70 mM sodium nitrate, 7 mM potassium chloride, 6 mM potassium phosphate, 5 mM magnesium sulfate, and Hunter’s trace elements) or complete media (CM; 1% glucose, 0.15% yeast extract, 0.15% casamino acids, 70 mM sodium nitrate, 7 mM potassium chloride, 6 mM potassium phosphate, 5 mM magnesium sulfate, 100X vitamin solution, and 1000 × Hunter’s trace elements solution) [41]. RPMI1640 medium (GIBCO BRL, Life Technologies, Woerden, The Netherlands) was used to study the effects of antifungal drugs in the broth media. Conidial spores of *A. fumigatus* were grown on MM solid media for three days at 37℃. A 5 ml of 0.01% Tween 80 solution was transferred to the surface of the colony and rubbed with a glass spreader. Conidial spores were filtered with autoclaved miracloth (475855, Merck KGaA, Darmstadt, Germany) and stored in 0.01% Tween 80 solution until use.

### Antifungal activity assay

To confirm the antifungal effect of drugs, resazurin assay was performed as reported previously [42]. Cells were grown on the PDA plate (0.4% potato infusion, 2% dextrose, 1.5% agar) for 48 hours at 37℃, and conidia were harvested using 0.85% saline with 0.01% Tween 20. The number of cells was counted using a hematocytometer. A 0.02% resazurin solution in distilled water was prepared and added to the 96-well plate at a final concentration of 0.002%. Caspofungin was prepared according to CLSI M38-A2 protocol and added to the well at the indicated concentration. Cells were inoculated at 2 × 10^5^/ml and incubated at 37℃ for 24 hours. Color changes were measured using a fluorescence reader, and growth inhibition was calculated. MIC50 and MEC50 values were calculated using “Quest Graph^TM^ IC50 Calculator” (AAT Bioquest, Inc.). To verify the antifungal effects of the compounds, each compound was serially diluted to the indicated concentration. All compounds were dissolved in DMSO. In the echinocandin class, drugs such as caspofungin (32343, Merck KGaA, Darmstadt, Germany), micafungin (SML2268, Merck KGaA, Darmstadt, Germany), and anidulafungin (SML2288, Merck KGaA, Darmstadt, Germany) were prepared at 4 mg/ml as stock solution, and the solutions were serially diluted to the lowest concentration. Amphotericin B (A2942, Merck KGaA, Darmstadt, Germany) and voriconazole (PHR1892, Merck KGaA, Darmstadt, Germany) were prepared with the 1 mg/ml stock solution and 720 μg/ml solutions, respectively, and serially diluted to the indicated concentration. Quinolone antibiotics such as ciprofloxacin (PHR1167, Merck KGaA, Darmstadt, Germany), nalidixic acid (97023, Merck KGaA, Darmstadt, Germany), levofloxacin (1362103, Merck KGaA, Darmstadt, Germany), norfloxacin (34058, Merck KGaA, Darmstadt, Germany), and ofloxacin (PHR1168, Merck KGaA, Darmstadt, Germany) were also prepared as indicated solutions for the growth assay. A total of 10^5^/ml conidial spores of *A. fumigatus* were inoculated into each well of a 96-well plate. The conidia were grown in 200 μl of RPMI1640 medium in the well for three days at 37℃. All experiments were performed according to CLSI protocols. A plate spotting assay was performed to check the growth of *A. fumigatus* and to confirm susceptibility to various chemicals. A total of 3 × 10^5^ spores were spotted on each plate and incubated for three or five days at 37℃ (Supplementary Figure S1).

### RNA sequence analysis

Total RNA of *A. fumigatus* was isolated using RNAiso Plus (Takara Bio Inc, Japan) reagent. RNA quantification was measured using ND-2000 Spectrophotometer (Thermo Inc., DE, USA), and RNA quality was assessed using an Agilent 2100 bioanalyzer with an RNA 6000 Nano chip (Agilent Technologies, Amstelveen, The Netherlands). Libraries were prepared from 2 μg of total RNA using the SMARTer Stranded RNA-Seq Kit (Clontech Laboratories, Inc., USA). The mRNA was isolated using a Poly(A) RNA Selection Kit (LEXOGEN, Inc., Austria). The isolated mRNAs were used for cDNA synthesis and shearing, following the manufacturer’s instructions. Indexing was performed using the Illumina indexes 1–12. The enrichment step was conducted by PCR. Subsequently, libraries were checked using the Agilent 2100 bioanalyzer (DNA High Sensitivity Kit) to evaluate the mean fragment size. Quantification was performed using the library quantification kit with a StepOne Real-Time PCR System (Life Technologies, Inc., USA). High-throughput sequencing was performed as paired-end 100 sequencing using HiSeq 2500 (Illumina, Inc., USA). mRNA-Seq reads were mapped using TopHat software to obtain the alignment file. The alignment files were also used for assembling transcripts, estimating their abundances, and detecting the differential expression of genes and isoforms using cufflinks. The expression level of the gene regions was determined using the fragments per kilobase of exon per million fragments (FPKM) method. The FPKM data were normalized based on the quantile normalization method using EdgeR within R (R development Core Team, 2016).

### Ergosterol extraction and HPLC analysis

Ergosterol was extracted from *A. fumigatus* as described [43] with a slight modification. *A. fumigatus* was cultured in 200 ml AMM containing drugs at the indicated concentration at 37°C, 220 rpm for 24 hours. The concentration of drugs treated in this experiment was 0.1% DMSO as control, 0.2 μg/ml voriconazole, 0.005 μg/ml caspofungin, and 0.005 μg/ml NE-E07. The cultured mycelia were harvested by filtration with miracloth and washed three times with sterile distilled water. Then, they were dried in an oven at 60°C and ground in a mortar. A 20 mg of dried cells was treated with 3 ml of 25% alcoholic potassium hydroxide and mixed by vortexing for 1 minute and incubated at 85°C in a water bath for 1 hour. A 25% alcoholic potassium hydroxide was prepared by adding 25 g of potassium hydroxide to 35 ml of H2O and then adding absolute alcohol to 100 ml and stored at 4°C. The cell lysate was cooled to room temperature, and sterols were extracted by adding 1 ml of sterile distilled water and 3 ml of hexane, and mixing by vortexing for 3 minutes. The mixture was incubated at room temperature until the hexane layer was separated. A 0.5 ml of the upper hexane layer was transferred to a new tube and evaporated in a fume hood at room temperature. The sample was stored in the dark at 4°C.

Before HPLC analysis, the pellets were dissolved in 100 µL of methanol and analyzed using an Acquity UPLC system (Waters, Milford, MA). The mixtures were separated using a UPLC BEH C18 column (2.1 × 100 mm, 1.7 μm, 0.3 ml/min) at 35°C with a mobile phase of water + 0.1% formic acid (A) and acetonitrile (B) and isocratic elution of 97% B (0–7 min) with a photodiode array detector. The extracted ergosterol peak was compared with standard ergosterol at 282 nm.

### Docking study

To characterize the binding sites and modes of the known inhibitor VT1161, NE-E07, and NE-B04 on the CYP51 binding site, VT1161, NE-E07, and NE-B04 were docked to the active site of CYP51, including the domains using Schrodinger. The starting conformation of ligands was obtained by the method of energy minimization with the Optimized Potentials for Liquid Simulations (OPLS) 2005 force field. The template for docking was derived from the crystal of CYP51 (5TZ1) [44], which was prepared using protein preparation wizard in Maestro 12.3 (Maestro Version 12.3.013, MMshare Version 4.9.013, Release 2020–1). For the docking study, we used a docking method with Glide 5.5. (Schrodinger Inc.) The Glide is based on grids for energy scoring and VT1161 matching. This Glide software was then used for the docking study of VT1161 and CYP51 heme binding site. The glide docking grid was prepared with default settings using VT1161 to define the location of the center of the grid. The grid boxes for docking were of the following dimensions: 25 × 25 × 25 Å^3^ (inner box: 10 × 10 × 10Å^3^); centered on the docked VT1161.

### Mouse survival assay

*A. fumigatus* conidia were grown on solid MM media for five days at 37℃. Conidia were harvested on the day of fungal infection using 0.9% saline containing 0.01% Tween 80. A five-week-old male BALB/C mouse was purchased (Doo-Yeol Biotech Co. Seoul, Korea) and incubated for one week to adapt to the environment. The body weight of all mice was between 18 and 20 g when immunosuppression was performed. For immunosuppression, mice were injected with 150 mg/kg cyclophosphamide four days before fungal infection, and the injection was continued at three-day intervals (−4,–1, + 1, + 4, + 7 days of infection). Cortisone (112 mg/kg) was injected once on the day before the fungal infection. Mice were anesthetized during the fungal infection using an inhalational anesthetic, isoflurane. A total of 5 × 10^5^ conidial spores were dropped intranasally; 1 mg/kg caspofungin was injected intraperitoneally after 24 hours from fungal infection; 5 mg/kg E7 was dissolved in 20% HP-β-CD, and the drug was administered by oral administration after 24 hours from fungal infection. Treatments were administered daily until the endpoint of the experiment. The mice were monitored, and their survival was recorded daily; 1 g/L tetracycline was administered to prevent bacterial infection. At the end of the experiment, all mice were killed using CO_2_ gas chamber according to the IACUC guidelines, Korea University. All animal experiments were performed in the Central Laboratory Animal Research Center, Korea University, Seoul, Korea. Experiments were repeated in duplicate. Statistical analysis between groups was evaluated using Log-rank test, and *P* values less than 0.05 were considered to indicate significance.

## Supplementary material

Online supplementary figure 1 NE-E07 does not potentiate the activity of the echinocandins against *C. albicans* and *C. neoformans*.

## Data Availability

All data generated from this work are included in the manuscript and its supplementary files.

## Abbreviations

AMM: *Aspergillus* minimal medium
IA: invasive aspergillosis
MOA: mechanism of action
