# Supplementary material for: The fluoroquinolone compounds potentiate the antifungal activity of the echinocandins against Aspergillus fumigatus
Source: Biosci Rep. 2025 Feb 4;45(2):BSR20250001. doi: 10.1042/BSR20250001 (PMC12096952; doi:10.1042/BSR20250001)
Supplement: Online supplementary figure 1 [file bsr-45-02-bsr-2025-0001-s001.docx]

**Fig. S1.** **NE-E07 does not potentiate the activity of the echinocandins against *C. albicans* and *C. neoformans*. (**A)Whether or not NE-E07 potentiates the activity of echinocandins against *C. albicans* was investigated. The overnight cultured cells were diluted to 0.01 at OD600 and 5μl of cells were spotted onto YPD plates treated with the indicated drug concentrations and incubated for 3 days. (B) And same experiment was performed against *C. neoformans*.

Fig. S1.

(A)

(B)
